# Antibiotic stewardship and antimicrobial resistance in conflict-affected Sudan: a situational analysis

**DOI:** 10.3389/fpubh.2025.1589290

**Published:** 2025-08-07

**Authors:** Megan Fletcher, Mei Trueba, Leena Al-Hassan

**Affiliations:** ^1^Department of Global Health and Infection, Brighton and Sussex Medical School, Brighton, United Kingdom; ^2^Centre for Infection and Antimicrobial Research (CINAMR), Department of Global Health and Infection, Brighton, United Kingdom

**Keywords:** antimicrobial resistance, antimicrobial stewardship, healthcare professional, conflict, Sudan

## Abstract

**Introduction:**

Antimicrobial resistance (AMR) is a global concern, the drivers and consequences of which are exacerbated by poverty and inequality, with Low- and Middle-Income Countries (LMICs) bearing the highest rates, particularly Sub-Saharan Africa. Armed conflict further exasperates problem, by damaging healthcare systems, limiting access to essential medicines, and increasing the use of antibiotics in uncertain environments. This study sought to explore healthcare professionals’ views on AMR and antimicrobial stewardship challenges before and during the conflict to investigate the impact of armed conflict on antimicrobial stewardship (ASP) in Sudan, a low-income country facing a humanitarian crisis exacerbated by ongoing violence.

**Methods:**

Data was collected via an anonymous qualitative online survey completed by healthcare workers with experience before and during the conflict and was analysed via thematic analysis.

**Results:**

Sudan is experiencing significant challenges due to its fragile healthcare system, poverty, and inequality. The results identify barriers to ASPs before and during the conflict, including lack of laboratory facilities, inadequate training, and the indiscriminate use of antibiotics. The findings reveal that the conflict has intensified the risk of AMR, with disrupted healthcare services leading to increased self-medication and reliance on broad-spectrum antibiotics.

**Discussion:**

The study underscores the urgent need for multi-faceted interventions that address immediate healthcare needs while implementing long-term strategies to control AMR in conflict-affected regions. Recommendations include enhancing healthcare infrastructure, improving diagnostic capabilities, and fostering collaborations among various stakeholders to ensure sustainable AMR prevention strategies.

## 1 Introduction

The rise in antimicrobial resistance (AMR) is a global concern. AMR contributed to 4.95 million deaths in 2019 and is estimated to cause 10 million deaths per year by 2050 if no action is taken (1, 2). According to the World Health Organisation (WHO), the drivers and consequences of AMR are exacerbated by poverty and inequality, and despite it affecting countries in all regions and at all income levels, Low- and Middle-Income Countries (LMICs) bear the highest rates, with the most significant rates being currently observed in Sub-Saharan Africa (1, 3). Research indicates that this is mostly due to weak health systems, limited diagnostic laboratory infrastructure to support robust surveillance, and unregulated and improper use of antibiotics (1, 3). Growing literature also highlights the significant impact that armed conflict has on the development of AMR. For example, population displacement has been seen to lead to infrastructure devastation, healthcare disruption, unsanitary conditions, and an increased need for invasive surgeries, all of which contribute to AMR (4). Another study examining the Iraq wars also noted that the deterioration of the healthcare system, misuse of antibiotics, and environmental contamination with heavy metals were significant factors driving AMR (5). Research also shows how armed conflicts, particularly in low- and middle-income countries, exacerbate challenges in AMR surveillance and control due to the lack of microbiology laboratories, antibiotic stewardship, and infection prevention programs (6). These studies collectively underscore the complex relationship between armed conflict and the rise of AMR (7, 8) due to damage to microbiology facilities preventing accurate bacterial identification (9), increased wound infections (10), forced migration (11), limited application of infection prevention and control (IPC), and key antibiotic blockages (12). Conflict, in turn, also hinders efforts in antibiotic stewardship programmes (ASP), aimed to coordinate strategies to optimise antibiotic access and use, which is one of the main selective pressures for the development of antibiotic resistance (ABR). Armed conflict impacts ASPs in a variety of ways, including increased self-medication as healthcare access is limited and the reliance on broad-spectrum antibiotic use among health professionals due to the lack of laboratory facilities (13, 14). Previous studies have shown high levels of ABR in conflict zones, such as in Médecins Sans Frontières (MSF) hospitals in Iraq and Jordan (8, 14, 15). Rising infection and ABR was also reported in Gaza, with a 300% increase in resistance to specific antibiotics seen in isolates from injured patients after the Great March of Return demonstrations, compared with non-injured patients (16).

Our research focuses on Sudan, a low-income country located at the crossroads of Sub-Saharan Africa and the Middle East. Sudan was one of the world’s poorest countries even before the conflict erupted between the Sudanese Armed Forces (SAF) and the Rapid Support Forces (RSF) in April 2023. Already before the conflict, the country faced a double burden of communicable and non-communicable diseases, high maternal mortality rates, in addition to widespread poverty, food insecurity, and various economic challenges, counted with a fragile health system, with low health indicators and disparities between regions (17). According to the data from the Global Burden of Diseases, in 2019, the country was also endemic to several Neglected Tropical Diseases, including leishmaniasis, trachoma, and schistosomiasis before the conflict, and bacterial sepsis was the leading cause of death in Sudan (17).

Long before the current conflict, a study published in The Lancet in 2005 highlighted the escalating concern of AMR in Africa, including Sudan, where over-the-counter antibiotics and self-medication were common (18). In 2017, the WHO’s Global Antimicrobial Resistance Surveillance System (GLASS) acknowledged that Sudan was one of the countries where AMR was becoming a growing concern (19). Since the ongoing conflict began in 2023, there has been widespread violence and instability (20), leading to the destruction of already weakened critical infrastructure, including hospitals and healthcare facilities, causing a significant humanitarian crisis. The conflict has also paralysed Sudan’s AMR efforts. In 2017 with support from international organisations, the Sudanese Ministry of Health began to take steps towards addressing AMR by developing national action plans (NAP) which launched in 2018 and guidelines aimed at improving the rational use of antibiotics (19). Sudan’s Ministry of Health participated in the WHO’s Global Action Plan on AMR and in 2017, the country was part of a regional meeting to address the rising threat of AMR across Africa (21). Sudan was at a critical point in addressing AMR, with some promising steps taken despite significant challenges, such as adopting a One Health approach that integrates human, animal, plant, and environmental health sectors in its NAP. Shortly before the conflict erupted, Sudan had also joined the GLASS and expanded its surveillance sites, reporting over 1,000 isolates/infections within a year. The Tailoring Antimicrobial Resistance Programs (TAP) pilot project was introduced to modify prescription practices among farmers, veterinarians, prescribers, dispensers, and antibiotics consumers in rural communities, resulting in a significant decrease in broad-spectrum antibiotic prescriptions (22).

The conflict in Sudan has not only intensified the need for medical care but also significantly increased the risk of ABR due to disrupted healthcare systems and the overuse of antibiotics. This is coupled with the fact that scarcity of essential medicines and other health system challenges further exacerbate an already critical situation by heightening the risks of drug-resistant infections (17). As of 2024, over 70% of Sudan’s hospitals have been damaged or rendered inoperable, severely hampering access to essential healthcare services (23). The conflict has also displaced millions, further exacerbating people’s food insecurity and limiting their access to clean water and sanitation. However, due to the scarcity of systematic data on antibiotic use, ASP and ABR rates, it is difficult to accurately measure how conflict has impacted AMR in Sudan (17).

Our study presented here departs from the hypothesis that the conflict in Sudan will exacerbate AMR. We present the results of a study that sought to understand healthcare professionals’ views on ABR and ASP challenges before and during the conflict to identify strategies able to improve ASPs and reduce ABR in Sudan and in other conflict-affected countries.

## 2 Methodology

### 2.1 Data collection

The data presented in this manuscript was part of a larger study investigating ASP and AMR within conflict-affected countries in the Middle East. An anonymous qualitative online survey was created using Qualtrics (Version 2024) and sent to individuals with healthcare experience before and during the most recent conflict in Sudan. The survey was open for 4 months from February to June 2024, and consisted of 39 close-ended and open-ended questions to capture information on respondent’s work role and experience, and on the practices and policies related to hospital infrastructure in microbiology and ASP prior to and during the conflict (Supplementary file 1).

Initially, potential participants were identified via our networks (as researchers working on AMR and in conflict-affected countries) and via key organisations with connections with researchers and healthcare professionals who have experience and/or are working with/in conflict-affected countries. Gatekeepers included: Doctors Without Borders (Médecins Sans Frontiers), College of Medicine, University of Baghdad (Iraq), The American University of Beirut (Lebanon), University of Khartoum (Sudan), University of Sherbrooke (Canada), University of Umea (Sweden), Rutgers University (USA). To maximise the recruitment of potential research participants, the survey link was also shared on X (Twitter) through the Brighton and Sussex Medical School (BSMS)’s communications teams. BSMS has a long tradition of collaborative research with health professionals working in Africa, many of which were based in Sudan and were also followers of the BSMS X. The pool of participants was then expanded via snowballing.

### 2.2 Inclusion and exclusion criteria

Upon clicking the survey link, potential participants were prompted to read a project information sheet (PIS) which described project aims, methods, inclusion and exclusion criteria for participation, and contract details for the research team in case they wanted to approach them with queries. No personal identifiable information was recorded, and only participants who provided consent were able to proceed to the survey. To be included, respondents must have been a healthcare professional (HCP) such as a doctor, nurse, laboratory technician, pharmacist, or healthcare director, aged 18 or over, and have been employed by national or international organisations within the last 20 years (from 2004 to June 2024). This was to ensure inclusion of a wide array of individuals with pre-conflict experience of healthcare work in Sudan, and to avoid including responses that may reflect the experiences of HCPs from the Sudanese Civil War which took place from 1983 to 2004.

### 2.3 Data analysis

Participants’ responses were extracted to an EXCEL file for analysis. Incomplete responses (all of which had provided information about the role and experience of the participants only) were removed from the final data analysis. The responses from research participants that did not meet the above-mentioned inclusion criteria were also removed before the start of the data analysis.

Data was manually analysed via thematic analysis, and included descriptive statistics on participant demographics. The qualitative data presented below details the role of the HCPs and years of experience. A pre-post conflict comparative approach was also used to investigate the perceived changes in AMR and ASP and the impact of armed conflict on ASP in Sudan.

### 2.4 Ethical approval

All ethical approvals were obtained from the Brighton and Sussex Medical School (BSMS) Research Governance and Ethics Committee before the start of any data collection (approval number ERA/BSMS9Z09/1/1).

## 3 Results

### 3.1 Research participants

A total of 67 potential research participants clicked on the survey link. From these, 18 did not read the PIS or closed the application before continuing to the next page and 17 did not provide consent. From the 32 potential research participants that provided consent, 12 filled out only the demographic information section of the survey. Ultimately, 15 individuals provided consent, fully completed the survey and were included in our study.

Ten women and 5 men completed the survey, aged between 26 and 35. Their professional experience included laboratory technicians (*n =* 5), doctor (*n =* 3), pharmacist (*n =* 6), nurse (*n =* 1), and were employed in a variety of facilities: Hospital (*n =* 8), central laboratory (*n =* 1), university (*n =* 1), pharmacy (*n =* 4), and primary care clinic (*n =* 1), with a range 6 months-13 years of experience (average = 4.65 years). Seven respondents (47%) worked immediately before the conflict started, 1 (6%) worked in Sudan 1–3 years before the conflict, 3 (20%) worked in Sudan over 3 years ago before the conflict started, and it was unclear on the times frames of 4 (27%) of respondents. Out of the total 15, 7 stated that they were working in Sudan during the conflict when the survey was conducted.

### 3.2 Thematic analysis

#### 3.2.1 Burden of antibiotic-resistant infections

All the survey respondents stated overall high rates of antibiotic-resistant infections (both prior to, and currently during, the conflict), with some respondents further explaining that they are attributable to “*irrational use of antibiotics*” (*n =* 2/15, pharmacists with 6 months and 7 years’ experience, respectively), lack of reliable microbiological data and policies to guide prescriptions. During the conflict, additional reasons were given which include lack of healthcare providers resulting in indiscriminate use of medications in general, and antibiotics in particular. Other reasons include the facilities not working due to the war, and fewer patients seen at hospitals due to displacement. 20% reported that the overall burden of ABR in their setting has increased since start of the conflict, whereas 26% (*n =* 4) said it remained the same. Notably, some (*n =* 7, 46%) said they did not know due to the disruption of the healthcare facilities and subsequent lack of data. One respondent stated perceiving a change in patterns of antibiotic-resistant infections, with increased incidence of Gram-negative bacteria since the conflict.

#### 3.2.2 Antibiotic availability and use

Already before conflict, participants reported that antibiotics were normally prescribed without a prescription, due to a lack of policies (4/15): *“… the antibiotics are dispensed without any rules or prescriptions”* (community pharmacist, 2010–2023). Without these policies, antibiotics were available over the counter: *“Irrational use of antibiotics, dispensing of antibiotics as OTC [Over the counter] drugs lead to high rate of resistance”* (community pharmacist, 2015–2022).

Also before the conflict, antibiotic supply issues were reported to be a barrier to ASP implementation by various informants, who mentioned that antibiotics were frequently out of stock relying in some instances on social media to find antibiotic stocks in the private market.

Over the counter and self-prescribed use of antibiotics is an ongoing AMR driver during the conflict, exacerbated by the broken-down healthcare infrastructure, lack of antibiotic availability, and the possibility of counterfeit antibiotics with poorer quality. “*The quantity increased but unfortunately the quality decreased*” (Laboratory Technician with 4 years experience).

#### 3.2.3 Increased demand of antibiotics due to reduced infection prevention and control (IPC)

Before the conflict, lack of IPC interventions was reported as a barrier to ASP implementation because antibiotics were needed to treat suspected infections. *“In the setting I worked in, it was under-resourced healthcare systems, insufficient practices in infection control, inadequate patient isolation, and a lack of sterile procedures, all of which contribute to the spread of antibiotic-resistant infections”* (hospital doctor, 2015–2018). This same respondent commented on the lack of IPC during the conflict: *“There is also an increased reliance on antibiotics as preventive measures in unsanitary conditions.”* This was supported by another hospital doctor (2015–2018): *“public health measures and monitoring are generally reduced, exacerbating the situation.”*

During the war, unsanitary conditions increase, public health measures decrease, traumatic wound infections increase, and without appropriate IPC, infections prevail and spread, subsequently leading to increased use to antibiotics. *“Lack of proper management of antibiotic-resistant infection during the conflict may increase the spread of resistant bacteria”* (hospital doctor, 2015–2019). This is further supported by a community pharmacist who also noted the role of forced migration and population density in safe areas as a contributing factor to increased infection transmissions and subsequent use of antibiotics: *“Because the number of people increased in one area where it considered safe. Accordingly, the infections and the use of antibiotics also will increase.”*

#### 3.2.4 Antibiotic stewardship programmes and hospital laboratory facilities

In relation to the availability of ASPs, only 27% reported an ASP was available before the conflict, but none reported any current availability of ASP during the conflict. Even before the conflict, respondents reported that a lack of priority of ASP due to other more immediate concerns (such as Malaria and other NTD) was hindering ASP implementation. Additionally, lack of ASP training was a perceived barrier to successful ASP implementation leading to lack of knowledge on local antibiograms and optimal antibiotic prescribing practices was noted by the respondents.

Some respondents reported operational microbiology and laboratory facilities before the conflict, which could perform antibiotic susceptibility testing with one individual stating: *“I was routinely and frequently consulted on antimicrobial selection based on susceptibility tests”* (hospital pharmacist, 2001–2006). This was supported by a hospital doctor with 1 years’ experience (prior to the conflict), who indicated that blood and urine cultures were commonly performed to test for the presence of bacteria before antibiotics were given. However, one reported that testing used older technologies compared to newer systems: *“routine microbiological tests were used (not advanced techniques) leading to delays in results and refraining of many doctors to request them”* (hospital pharmacist, 2018–2020). On the other hand, some respondents reported inequality in microbiology access even prior to the conflict, where laboratory tests performed either by private laboratories outside of the hospitals, due to fully functional microbiology diagnostics only being present in large tertiary hospitals in the big cities. This was attributed several factors, such as resource limitations and lack of prioritisation of microbiological diagnostics, and the reliance on broad-spectrum antibiotic coverage regardless of culture and sensitivity results.

Some respondents also commented that microbiology test costs were a perceived barrier to ASP implementation even before the conflict: *“The expense of test and the low socioeconomic status are the big challenges”* (community pharmacist, 2010–2023). This is supported by another community pharmacist (2015–2022): *“Because socioeconomic status (of the patient) is the first factor that is usually considered by healthcare providers.”* As well as pharmacists, a hospital laboratory technician with 4 years’ experience, commented on the cost being a barrier: *“The budget of the test.”* However, a doctor (2004–2015) commented that the barrier was a general healthcare funding issue rather than individual socioeconomic status: *“Lack of funding.”* However, no respondents commented on financial barriers during the conflict.

#### 3.2.5 Healthcare access and human resources

Prior to the conflict, scarce and damaged facilities and challenges related to the cost of testing, as well as lack of HCP’s knowledge about the need for microbiology testing emerged as an important issue. It was reported that HCPs did not believe that microbiology and AST were important before prescribing antibiotics: *“Most doctors did not believe testing was necessary or useful”* (hospital pharmacist, 2001–2006).

This was similarly reported by respondents during the conflict: *“The use of high potency antibiotic without cultures”* (doctor with 1 year of experience)*. “Because of the lack of health care providers, the people will used antibiotic without any counselling from doctors or pharmacists*” (community pharmacist, 2010–2023). This was supported by another community pharmacist with 6 months of experience: *“Because, for example poor people who are affected by this war can no longer offer the fees of seeing a doctor so they share their antibiotics with neighbours instead.”*

When asked about operational capacity the microbiology and laboratory facilities during the conflict, respondents noted the conflict-induced destruction of healthcare facilities and laboratories: *“The facility was not working during the war”* (hospital laboratory technician currently working with 8 years’ experience). This was supported by a hospital nurse with 5 years’ experience: “*There are no facilities for microbiology testing. All the hospitals are destroyed*.” In addition to significant destruction of key facilities, looting and safety issues affecting laboratory operations during the conflict were also raised by the respondents. Another issue raised is the forced migration of HCP due to the conflict, affecting the adequate operations of the healthcare system as a whole: *“Migration of the specialists and absence of the fund to reopen the facilities”* (hospital doctor with 1 year’ experience).

## 4 Discussion

With this study we sought to understand healthcare professionals’ views on ABR and ASP challenges before and during the conflict in Sudan to identify strategies to improve ASPs and reduce ABR in Sudan, and in other conflict-affected countries more widely. The key barriers are summarised in Table 1.

**Table 1:** Summary of reported barriers to antimicrobial stewardship before and during the conflict.

Barriers to ASP implementation before the conflict	Barriers to ASP implementation during the conflict
Lack of infection, prevention and control	Unsanitary living due to forced migration
Lack of laboratory facilities	Destruction of laboratories
Lack of training	Quality of antibiotics decreased
Cost of microbiology tests	Blockages in supply chain
Supply chain barriers	Migration of specialists

Much of Sudan’s history has been marked by internal armed conflicts, regional inequities and a fragile state, especially in the healthcare sector (18, 23). The latest conflict erupted in April 2023, currently labelled as the worst conflict-induced humanitarian crisis globally, with the world’s largest internal displacement crisis, acute hunger and the looming threat of famine (24, 25). The war has devastated Sudan’s healthcare infrastructure, leading to closures and disruptions in medical services, particularly in war-affected areas. Already prior to this war, Sudan suffered from a fragile healthcare system which was underfunded and fragmented, with >95% of the population having to pay out-of-pocket for healthcare services (25–27). Public health facilities are primarily funded by the government (and external aid agencies) and aim to provide essential health services to the population including any medical tests required by the patient. However as evidenced in our results and other studies, even prior to the conflict, there were discrepancies in availability and access to laboratory facilities (i.e diagnostic microbiological testing) with some respondents stating it was operational, while others said it was commonly outsourced and only available in large tertiary hospitals (26). Furthermore, the socioeconomic status of patients was a major factor in the delivery of the healthcare service, determining what tests can be affordable for the patients. Consequently, HCP prescribed (affordable) broad-spectrum antibiotics to avoid charging the patient for a microbiology test (which should be part of the public health “package”).

Before the conflict, the country already faced significant critical shortages in its health workforce, with the density of physicians, nurses and midwives being estimated to be 3.6, 14 and 9.1 per 10,000 people, respectively, far below the WHO minimum threshold density of 22.8 health professionals (23). Despite these challenges, evidence suggests that Sudan was making steady AMR progress. Some hospitals, mainly those located in urban centres like Khartoum, were beginning to implement hospital-based ASPs aimed to optimise the use of antibiotics through better prescribing practices and improving diagnostic accuracy (26). However, as noted in the WHO’s 2019 report on antimicrobial resistance in Africa, due to various and overlapping logistical and financial challenges the implementation was not widespread, and the quality of these programmes was uneven across the country (28).

Literature suggests that in Sudan, ASP availability was scarce prior to the conflict mainly due to conflicting priorities, inadequate training, and insufficient knowledge of local antibiograms among other key aspects of AMR and infection control (26). These, in turn, harden the implementation of ASP during emergency situations like the current conflict, in an-already-fragmented healthcare sector. This is a significant concern for controlling ABR, as the absence of IPC and ASP means antibiotics are being used indiscriminately without proper guidance or monitoring, which is augmented by the destruction of 70% of public and private healthcare facilities in war-affected states, neither IPC or ASP are given priority, as the remaining ones are overwhelmed by the influx of people seeking care, many of whom are internally displaced (22).

In a study conducted in 2020 prior to the eruption of the ongoing conflict, 74% of healthcare workers stated no access to IPC or ASP materials, and infrequently receive relevant IPC training (26). For years, the Sudanese workforce has been depleted by severe brain drain, with low staff retention and high emigration of health-care workers, driven by political instability, low wages, and poor-quality training opportunities for many decades (29). As shown in our results, the conflict has only weakened further the available human resources, together with the healthcare facilities and the availability of key antibiotic and quality measures (26). The forced migration of many trained HCP is hindering the operational capabilities of the healthcare system as a whole.

Before the war, the country’s health profile was defined by a dual burden of disease—largely communicable diseases, which were marked by frequent outbreaks, alongside non-communicable diseases (NCDs). The war has not only exacerbated Sudan’s already fragile health situation but has also introduced new challenges, including conflict-related injuries and increased risk of ABR (30, 31). The shift from a dual to a quadruple burden of disease has resulted in health service demands that far exceed the capacity of the country’s weak, under-resourced and damaged healthcare system (30, 31). The conflict has significantly worsened the health profile of Sudan while simultaneously reversing the progress made in health system strengthening, AMR control, and ASP.

More research is needed to understand how conflict impacts ASP (14). In a study by Médecins Sans Frontières (MSF) in the Middle East document high levels of ABR, yet the literature lacks studies that accurately examine ABR over the course of conflicts, particularly due to the scarcity of microbiological surveillance. This is further exacerbated by global actions plans (GAPs) not accounting for emergencies such as conflict (32). In 2015, the WHO set out the GAP to reduce the global burden of ABR which includes five key areas: education, surveillance, improving water and sanitation access, optimise antibiotic use and research investments (32–34), but excludes conflict prevention or management. The lack of inclusion of conflict considerations in current action plans means that, as we have shown, it healthcare professionals struggle to access the necessary resources, knowledge and support available to reduce ABR during but while rebuilding post-conflict. Understanding the barriers and challenges to implement an ASP before and during conflict can help ensure ABR is kept under control even during conflict by explaining what is needed.

In the context of Sudan, any future plan to address AMR, the Sudanese health system must incorporate a long-term and appropriately resourced workforce strategy that covers training and incentives for retention of health-care professionals and administrative staff.

### 4.1 Reflections and study limitations

Researchers have raised concerns regarding the limited understanding of the relationship between ABR and conflict (8). A key strength of this study is its collection of first-hand accounts of ABR and ASP by health professionals working in a resource scarce country before and during a conflict, an approach that, to our knowledge, has not been conducted. In so doing, we have provided valuable insights into the experiences of HCPs in relation to AMR before and during conflict, as well as conflict-specific challenges to implementation which could guide future policy recommendations for individual countries and globally. We, however, recognise some limitations which may impede the generalisability or external validity of our findings: the breath of the presented data is limited, as it only encompasses one country and a relatively small sample. While there can be bias in all types of research (35), our research involves self-reported risks, including recall bias which refers to the potential for participants to inaccurately recall current and past events or experiences. While we recognise these potential risks, our research nevertheless brings important insights that align with those of other similar studies, and also brings evidence-based recommendations for conflict-specific challenges in relation to antimicrobial resistance. Due to the current political situation in Sudan, it was not possible to complement the survey data with key interviews which would have strengthened the results. In addition, the survey was in English language only, which is not the first language of people living in Sudan. Medicine and other health-related subjects are solely taught in English at Higher Education settings in Sudan, and therefore the use of English language was deemed appropriate. We however recognise that this research choice may have negatively influenced the number of survey responses.

## 5 Conclusion

Our study highlights the complex intersection of conflict, healthcare, and antibiotic resistance, showing how war exacerbates existing public health challenges. There is a need for multi-faceted interventions that address both immediate healthcare needs and long-term strategies for controlling ABR. To effectively mitigate ABR in conflict-affected areas we recommend multisectoral collaborations involving healthcare providers, governmental bodies, non-governmental organisations (NGOs), the private sector, and local communities enabling the sharing of resources, knowledge, and best practices, ensuring that ABR prevention strategies are both context-specific and sustainable. Investment in educational programmes that target healthcare workers, community leaders, and the general public will help instil long-term behaviour changes, which are vital for reducing the burden of infections and ABR. Furthermore, targeted investment in healthcare infrastructure, diagnostic technologies, and antimicrobial stewardship programmes will strengthen the response to ABR in these vulnerable settings.

A multisectoral approach, incorporating the Global Action Plan’s objectives into the context of conflict-affected areas and tailoring them to the specific local challenges, we can ensure that efforts to combat AMR are more coherent, strategic, and effective.

## Data Availability

The raw data supporting the conclusions of this article will be made available by the authors, without undue reservation.
