# Supplementary material for: Antibiotic stewardship and antimicrobial resistance in conflict-affected Sudan: a situational analysis
Source: Front Public Health. 2025 Aug 7;13:1589290. doi: 10.3389/fpubh.2025.1589290 (PMC12367764; doi:10.3389/fpubh.2025.1589290)
Supplement: Supplementary file 1 [file Supplementary_file_1.docx]

Antibiotic stewardship in Middle Eastern conflict affected countries: situational analysis and potential solutions.

1. What is your age?

2. What is your gender?

- Female
- Male
- Other
- Prefer not to say

| Please note that all questions in this survey relate to Middle Eastern countries which have been either directly or indirectly affected by conflict/war. **Directly** is here defined as the country experiencing war, whilst **indirectly** relates to countries experiencing forced movement of people due to the conflict in neighbouring countries.   - Please base your following answers on **one** specific Middle Eastern country affected by conflict. If you have experience working in various countries, please feel free to complete multiple surveys, one for each country you have worked in. Thank you for your assistance with this. |
| --- |

3. Which county are you basing your answers on?

- Iraq
- Sudan
- Jordan
- Lebanon
- Syria
- Palestine
- Yemen
- Other (please specify):

4. In this country, what type of healthcare facility did you work within?

- Hospital
- Clinic
- Field hospital
- Other (please specify):

5. Are you currently working there?

- Yes
- No

6. If you answered No to question 5, could you please specify when you worked there?

7. How long did you/have you worked there?

8. What is/was your job title when working here? (Please select the role that most fits your job title).

- Doctor
- Nurse
- Pharmacist
- Laboratory technician
- Health Care Assistant
- Infection, Prevention and Control
- Administration
- Health care policy maker

**Antibiotic resistant infections**

9. In your opinion, what were the rates of antibiotic resistant infections in your country of work before the conflict started:

- Very high
- High
- Medium
- Low
- Very low
- Not applicable
- I do not know

Please justify your answer

10. In your opinion, what were the rate of antibiotic resistant infections during the conflict.

- Very high
- High
- Medium
- Low
- Very low
- Not applicable
- I do not know

Please justify your answer

11: In your opinion, what were the rate of antibiotic resistant infections 12 months after the conflict:

- Very high
- High
- Medium
- Low
- Very low
- Not applicable
- I do not know

Please justify your answer

12. In your opinion, did you see a noticeable change in antibiotic resistant infections during or post conflict?

- Increase
- Decrease
- Stayed the same
- I do not know
- Change in type of infections

13. If you answered increase/decrease to question 12, what do you think were the contributing factors to the change in antibiotic resistant infections?

14. Did you see a change in the types of antibiotic resistant bacterial infections seen? i.e more Gram-negative vs Gram-positive bacteria? Please justify your answer

**Microbiology diagnostics and susceptibility testing**

15. Before the conflict, were the microbiology diagnostic(s) facilities operational to identify bacterial infections and perform antibiotic susceptibility testing?

- Yes
- No
- I do not know
- Not applicable

Please explain your reasoning

16. In your opinion, what were the main challenges to bacterial and antibiotic susceptibility testing before the conflict?

17. During the conflict, were the microbiology diagnostic(s) facilities operational as before the conflict)?

- Yes
- No
- I do not know
- Not applicable

18. In your experience, what were the challenges to microbiology diagnostics during conflict?

19. After the conflict, were the microbiology diagnostic(s) facilities operational as before the conflict)?

- Yes
- No
- I do not know
- Not applicable

20: In your opinion, what are the challenges to microbiology diagnostics after conflict?

**Antimicrobial stewardship**

The World Health Organization defines antimicrobial stewardship as tasks taken to educate and support healthcare professionals to promote correct antibiotic prescriptions and use. This is the definition used here.

**Prior to conflict**

21. Before the conflict, were antibiotics available and accessible?

22. Before the conflict, was there an antimicrobial stewardship policy in your work facility?

- Yes
- No
- I do not know
- Not applicable

If appropriate, please let us know your opinion on this policy and its application before the conflict:

23. If you answered yes to question 22, could you let us know if the policy was available and accessible in the place you worked?

- Yes
- No
- I do not know
- Not applicable

If you answered no or I don’t know, could you please let us know why?

24. In your opinion, what were the main challenges to antimicrobial stewardship prior to conflict?

**During conflict**

25. In your experience, are/were antibiotics available and accessible during the conflict?

26. Did you notice a change in access to antibiotics in comparison to before the conflict?

- Yes
- No
- I do not know
- Not applicable

Please clarify:

27. To your knowledge did/does the facility you worked in have an antimicrobial stewardship policy during the conflict?

- Yes
- No
- Not sure
- Not applicable

Please explain your reasoning for your answer

28. In your experience, how did the antimicrobial stewardship strategy change with the conflict?

29. In your opinion, is it possible to implement antimicrobial stewardship at times of conflict in this country?

- Yes
- No
- I do not know

Please justify your answer.

30. In your opinion, what are or were the main challenges of antimicrobial stewardship during conflict in your facility?

31. In your opinion, how could these challenges best be addressed?

**After conflict**

32. In your experience, are/were antibiotics available and accessible after the conflict?

33: Did you notice a change in antibiotic access in comparison to during the conflict?

- Yes
- No
- I do not know
- Not applicable

Please explain your reasoning

34. To your knowledge did/does the healthcare centre have an antimicrobial stewardship policy after the conflict?

- Yes
- No
- I do not know
- Not applicable

If appropriate, please tell us more about this post-conflict antimicrobial stewardship policy.

35. In your experience, how did the antimicrobial stewardship policy change after conflict?

36. In your experience, what are the main challenges of achieving antimicrobial stewardship after conflict?

37. In your opinion, how could these challenges best be addressed.

38. In your experience, do you think conflict provisions should be included in global and national antimicrobial stewardship programmes?

- Yes
- No
- I do not know

Please justify your answer

39. Is there anything that you feel is important for us to know?
